# Correction: Stepwise stroke recognition through clinical information, vital signs, and initial labs (CIVIL): Electronic health record-based observational cohort study

**DOI:** 10.1371/journal.pone.0243480

**Published:** 2020-12-03

**Authors:** Sung Eun Lee, Mun Hee Choi, Hyo Jung Kang, Seong-Joon Lee, Jin Soo Lee, Yunhwan Lee, Ji Man Hong

In the Funding statement, the grant number from the funder Korea Centers for Disease Control and Prevention is listed incorrectly. The correct grant number is: 2020-ER6303-00.
