# Supplementary material for: Stepwise stroke recognition through clinical information, vital signs, and initial labs (CIVIL): Electronic health record-based observational cohort study
Source: PLoS One. 2020 Apr 15;15(4):e0231113. doi: 10.1371/journal.pone.0231113 (PMC7159200; doi:10.1371/journal.pone.0231113)

Supplementary figure 1. The Korean version of EMR based matrix for stroke suspicious patients. (Cubic S model)

This model was made up 3 domains of time, body spatial, and symptoms: Sudden, Side, Symptoms.

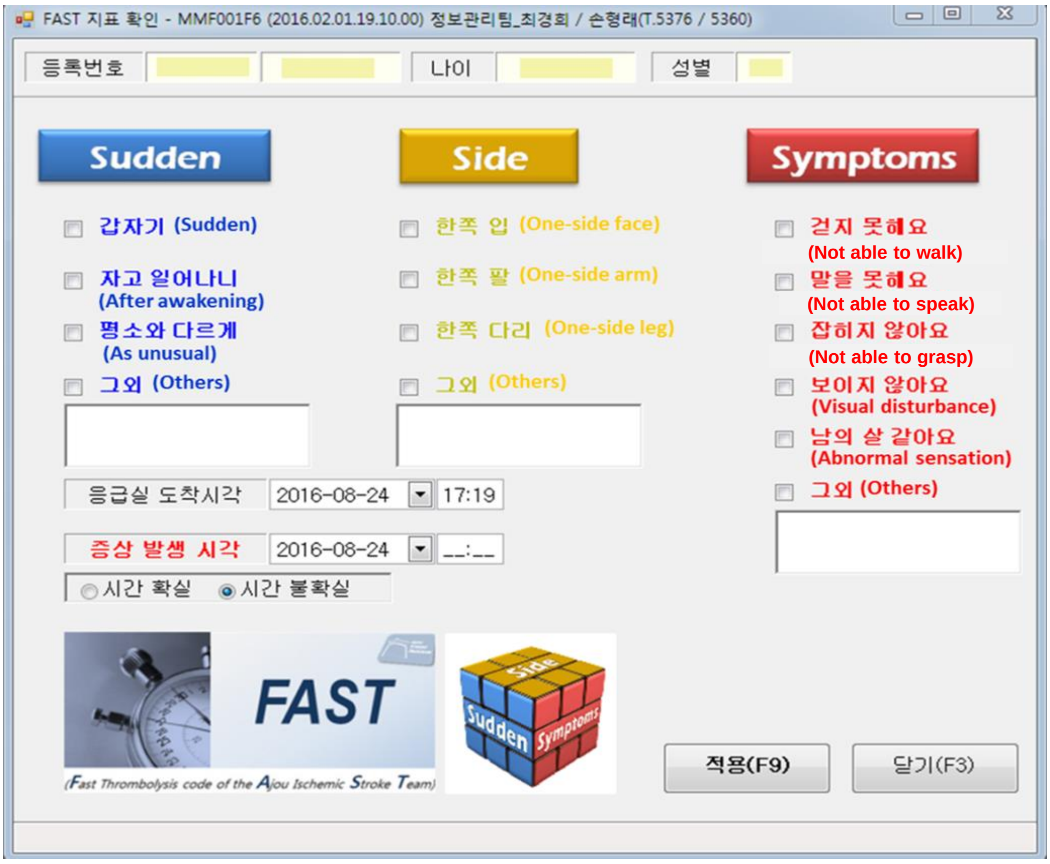

Supplement: S1 Fig — (Cubic S model) (PDF) [file pone.0231113.s003.pdf]
