# Supplementary figures and images for: Stepwise stroke recognition through clinical information, vital signs, and initial labs (CIVIL): Electronic health record-based observational cohort study
Source: PLoS One. 2020 Apr 15;15(4):e0231113. doi: 10.1371/journal.pone.0231113 (PMC7159200; doi:10.1371/journal.pone.0231113)

Supplementary figure 2. Flow diagram of 1,621 suspicious stroke patients.

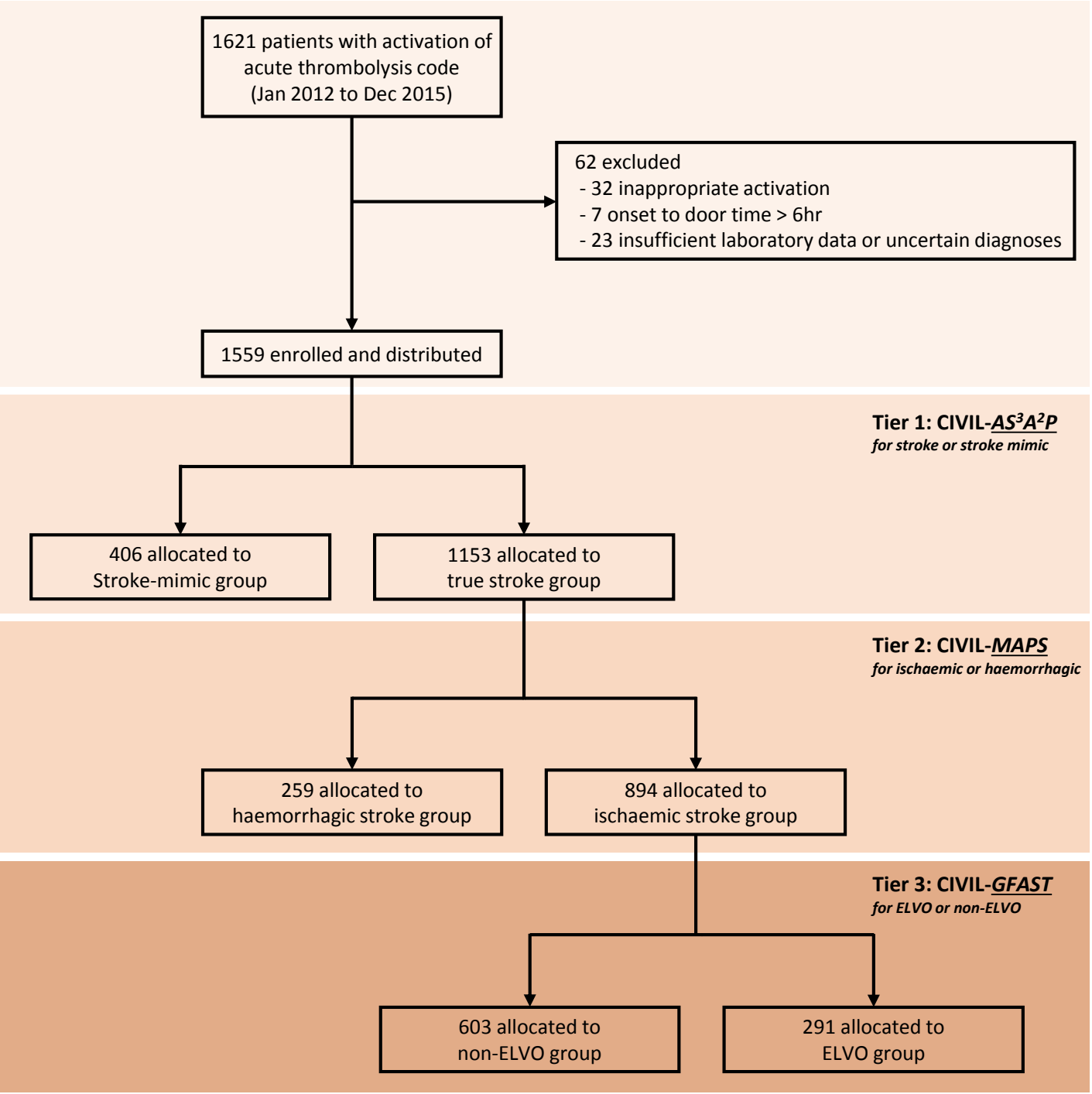

Supplement: S2 Fig — (PDF) [file pone.0231113.s004.pdf]
