# Supplementary material for: Stepwise stroke recognition through clinical information, vital signs, and initial labs (CIVIL): Electronic health record-based observational cohort study
Source: PLoS One. 2020 Apr 15;15(4):e0231113. doi: 10.1371/journal.pone.0231113 (PMC7159200; doi:10.1371/journal.pone.0231113)

Supplementary figure 3. Receiver-operating characteristic (ROC) curve and corresponding area under the curve (AUC) statistics of the CIVIL scoring system.

In step 1, performances of previous recognition systems (Cincinnati Prehospital Stroke Scale [CPSS], Los Angeles Prehospital Stroke Screen [LAPSS], and Recognition Of Stroke In the Emergency Room [ROSIER] system) were compared with the CIVIL system. In step 2 and 3, performances of the CIVIL system were presented.

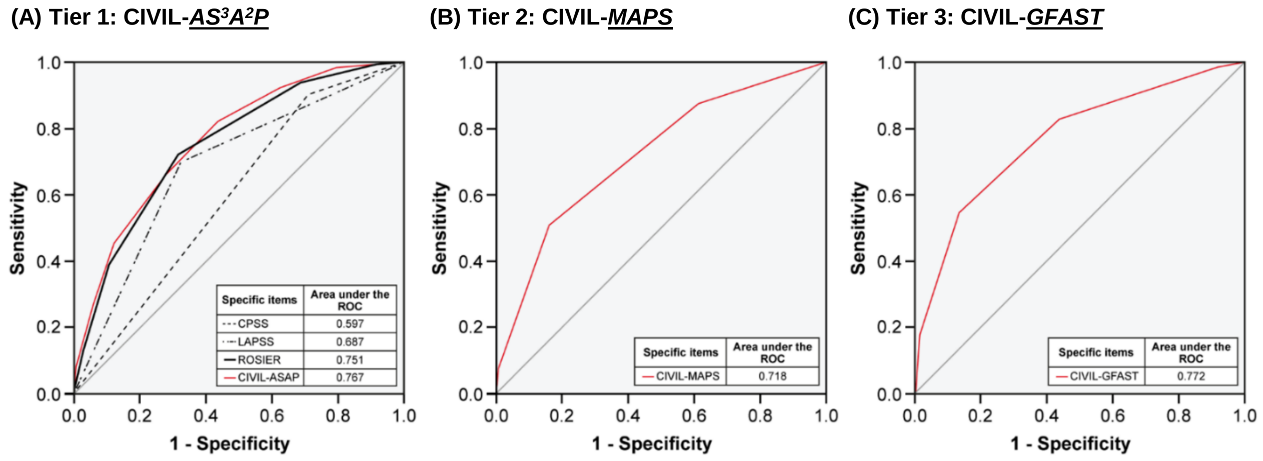

Supplement: S3 Fig — (PDF) [file pone.0231113.s005.pdf]
